# Aging-in-place preferences and institutionalization among Japanese older adults: a 7-year longitudinal study

**DOI:** 10.1186/s12877-022-02766-5

**Published:** 2022-01-21

**Authors:** Takeshi Nakagawa, Taiji Noguchi, Ayane Komatsu, Masumi Ishihara, Tami Saito

**Affiliations:** National Center for Geriatrics and Gerontology, Obu, Japan

**Keywords:** Community-dwelling adults, Decision-making, Hospitalization, Long-term care, Nursing home admission, Older adults

## Abstract

**Background:**

In Asia, where autonomous decision-making is not well accepted, little is known about whether and how individuals’ preferences are considered when deciding where they receive care. This study examined whether individuals preferring to age in place if confined to bed were less likely to be institutionalized, using longitudinal data of Japanese older adults.

**Methods:**

We analyzed nationally representative data of 1,290 community-dwelling older adults aged 70 and above. Baseline data were collected in 1999, shortly before the long-term care insurance system was introduced. The outcome was measured as self- or proxy-reported years of institutionalization over seven years. The explanatory variable was whether individuals preferred to age in place if they were confined to bed. Participants were asked about their desired place of care (facility, home, or other) if confined to bed. Covariates were sociodemographic and health-related factors. We used Cox proportional hazards models and calculated hazard ratios (HRs) with 95% confidence intervals (CIs) to evaluate the association of aging-in-place preferences if confined to bed with institutionalization. We applied multiple imputation to deal with missing data.

**Results:**

Seventy-eight respondents (6.0%) were institutionalized during the follow-up period. Compared to individuals preferring to reside in long-term care facilities if confined to bed (48.7%), those preferring to stay in their homes (39.6%) were less likely to be institutionalized, even after adjusting for relevant covariates (HR = 0.47, 95% CI 0.27–0.79 for model 1 including residential status; HR = 0.45, 95% CI 0.27–0.76 for model 2 including marital status and co-resident children).

**Conclusions:**

Our findings suggest that individuals’ aging-in-place preferences tend to be considered under the long-term care insurance system. Individuals’ preferences should be shared with families and clinicians when deciding the place of care.

**Supplementary Information:**

The online version contains supplementary material available at 10.1186/s12877-022-02766-5.

## Background

Aging-in-place refers to the preference to live in one’s own home. The vast majority of older adults are known to prefer to age in place. In 2018, more than 90 percent of Japanese adults aged 65 and above preferred to live at home, and more than half of them also preferred to die at home [1]. In fact, however, only 13.7% died in one’s own home [2]. Therefore, older adults and families along with clinicians and policymakers need to understand how individuals’ aging-in-place preferences are considered when determining whether to stay at home versus moving to a nursing home or a hospital.

Previous research has examined predictors of institutionalization (i.e., nursing home admission and long-term hospitalization) among community-dwelling older adults. Poor health, including physical and cognitive impairment, and sociodemographic factors, such as increasing age and living alone, are predictive of institutionalization, according to meta-analytic and systematic reviews [3, 4]. These health-related and sociodemographic factors are relatively consistent predictors of institutionalization in Japanese older adults as well [5–10]. However, few studies have directly tested whether the individuals’ aging-in-place preferences are considered when deciding whether to stay in one’s own home.

The limited evidence across the globe for the association between individual preferences and institutionalization may be partly because most studies have been conducted in individualistic cultures, such as North America, emphasizing individual autonomy and taking for granted that individuals have the right to make autonomous decisions [11, 12]. In contrast, some researchers and clinicians have recognized that individual preferences toward autonomous decision-making vary within and across cultures [11, 12]. Specifically, in collectivistic cultures, such as Asia, decisions are shared and made by individuals, families, and clinicians. As a result, autonomous decision-making has been less accepted in Japan, where families and clinicians traditionally made medical decisions without consulting the individuals [13]. Therefore, it would be particularly important in collectivistic cultures to examine to what degree individual preferences are considered when deciding the place of care.

Empirical studies have shown that individuals perceive families and clinicians to have crucial roles in treatment decision-making in Asia, including Japan [14–16]. In contrast, evidence remains scarce concerning whether and how individuals’ preferences are considered when deciding where they receive care. As an exception, an earlier study of Japanese older adults with severe disabilities suggests that individuals preferring to age in place live in their homes for a longer period [17]. However, one of the limitations is that the study asked care managers, but not older adults, about individual preferences. To better understand the role of individual preferences in care decision-making, this study aimed to examine whether individuals preferring to age in place if confined to bed were less likely to be institutionalized among Japanese older adults.

## Methods

### Participants and procedure

We used data obtained from the National Survey of the Japanese Elderly (NSJE), a nationally representative survey of older Japanese aged 60 and above. The NSJE started in 1987, and the participants were interviewed every three to four years until 2006. In 1999, people aged 70 and above were recruited shortly before the long-term care insurance system was introduced in 2000, and 1,635 people participated in the baseline survey (response rate = 81.8%). The participants were followed up in 2002 and 2006. We thus used the seven-year three-wave longitudinal data for the subsequent analysis. The detailed methodology, including the research design and the response rates, is available on the website of the NSJE [18].

**Fig. 1:**
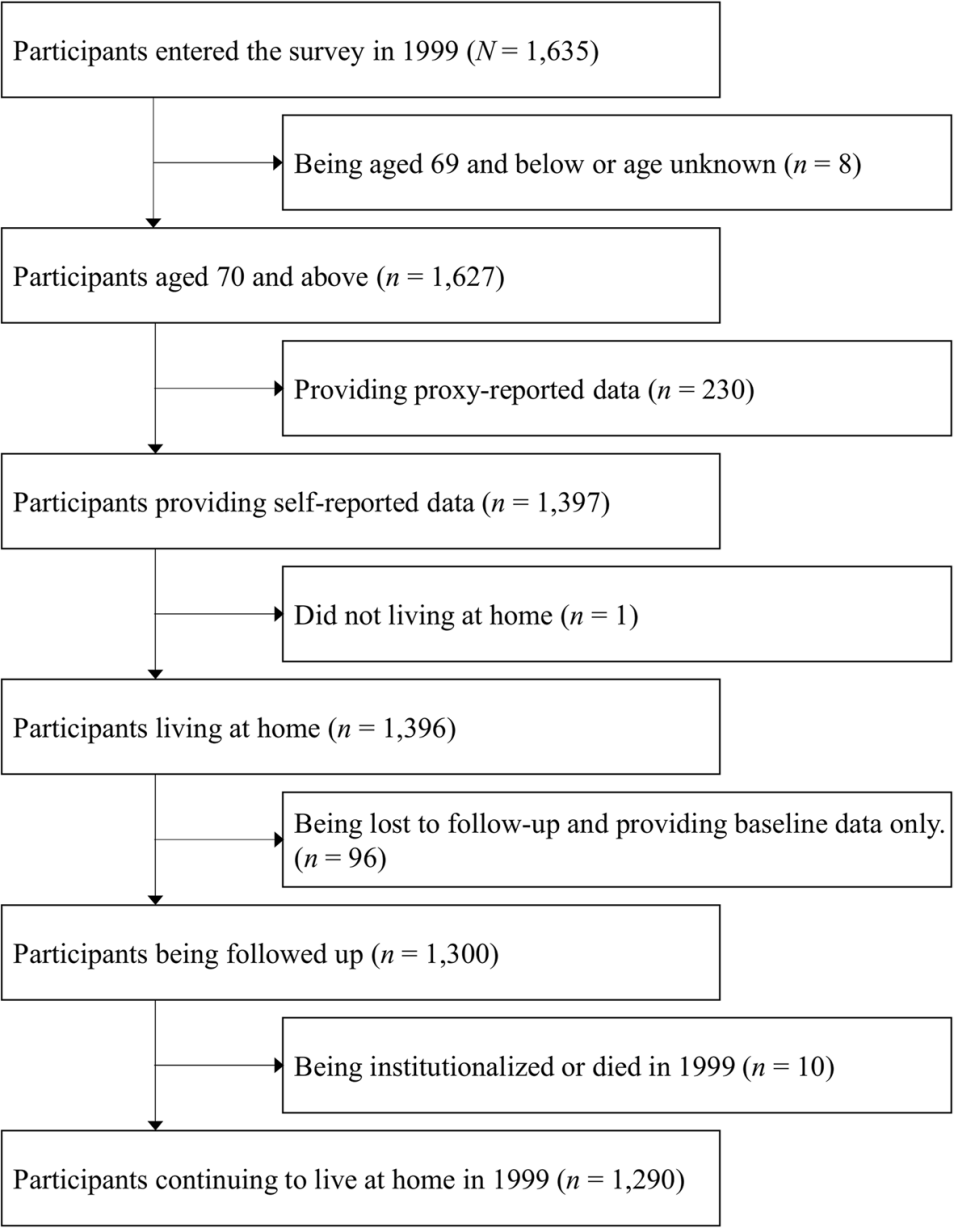
The sample selection procedure

The sampling procedure is illustrated in Fig. 1. The selection criteria were (i) 70 years old and above; (ii) self-reported data provided at the first survey; and (iii) living at home at baseline. The exclusion criteria were (iv) being completely lost to follow-up (i.e., those whose timing when they dropped out was not available), and (v) admission to a long-term care facility or died in 1999 just after the first participation. As a result, 1,290 respondents were selected for this study.

### Institutionalization

The outcome was measured as self- or proxy-reported years of institutionalization. In this study, we created a composite outcome by defining institutionalization as admission to long-term care facilities and long-term hospitalization. Since the long-term care insurance system was enacted in 2000, older adults could use a wide range of care services, including long-term care facilities. Then, time to institutionalization was calculated as the year of the baseline survey (1999) to the year of the last follow-up survey (2006) or the time when the participants were institutionalized, dropped out, or died. Residence status, including survival, was obtained and verified through the official residential registry of each municipality where the participants resided. The registries constitute addresses of all residences in alphabetical order.

We note that the temporary absence, such as usage of short-stay services and hospitalization for a short period (defined as less than one month), was considered as community-dwelling. In addition, residential places, such as low-cost social welfare facilities and retirement homes, were not regarded as institutionalization because those residents were assumed to live relatively independently.

### Aging-in-place preferences

The explanatory variable was whether individuals preferred to age in place if they were confined to bed. Participants were asked about their desired place of care if they were confined to bed. Then, respondents chose one of the seven options: hospital, nursing home, home with informal caregivers, home with formal caregivers, retirement housing, other, and do not know. In this study, we classified the responses into three preferences (i.e., facility, home, and other): (i) facility included two options (hospital and nursing home); (ii) home included three options (home with informal caregivers, home with formal caregivers, and retirement housing); and (iii) other included the remaining two options (other and do not know). We created two dummy variables with the facility as the reference category.

### Covariates

Considering predictors of institutionalization [3, 4] and preferences for care [19], we included several sociodemographic and health-related variables as covariates: age at the first survey, gender (0 = *male*, 1 = *female*), years of education, perceived financial status (0 = *extremely difficult* to 4 = *not at all difficult*), living arrangement (0 = *living alone,* 1 = *living with others*), non-coresident children (0 = *no*, 1 = *yes*), and physical and cognitive function. To check the robustness of results on family networks, we also considered marital status (0 = *not married*, 1 = *married*) and co-resident children (0 = *no*, 1 = *yes*), instead of living arrangement.

Physical function was indexed as activities of daily living (ADLs). ADLs were assessed using ten activities (e.g., taking a bath, getting dressed, and moving in and out of bed), answered on a scale ranging from 0 = (*cannot)* to 4 = (*not difficult)*. We calculated the summary score, ranging from 0 to 40. A higher value represents better physical function.

Cognitive function was assessed using the Short Portable Mental Status Questionnaire (SPMSQ) [20, 21]. Nine items were measured in the NSJE (e.g., memory, time and place orientation, and serial calculation). The number of correct answers was summed and used as the indicator of cognitive function. The score ranged from 0 to 9. A higher score indicates better cognitive function.

### Statistical analysis

We first reported descriptive statistics by institutionalization and intercorrelations among the study variables. Next, we used Cox proportional hazards models and calculated hazard ratios (HRs) with 95% confidence intervals (CIs) to evaluate the association of aging-in-place preferences if confined to bed with institutionalization. The proportional hazard assumptions were graphically assessed using the Kaplan–Meier methods and tested using Schoenfeld residuals.

The percentage of respondents having missing information on the explanatory variable or covariates was 29.9%. To mitigate potential bias due to missing data, we applied multiple imputation. The method yields unbiased estimates and standard errors when the missing at random assumption is satisfied [22]. The imputation model included the explanatory variable and covariates at baseline. According to the guideline, we conducted 30 imputations, equivalent to the percentage of incomplete respondents. The underlying Markov chain was iterated ten times for each imputation. Then, to check the imputation model, we tabulated summary statistics of the observed and imputed data [23]. Similar means and standard deviations between the two data indicate that the imputation model is well specified. Finally, results were aggregated across the analyses to derive summary statistics by standard procedures [22]. All statistical analyses were conducted using SPSS, version 28 (IBM Corp., Armonk, NY, USA).

## Results

### Descriptive statistics and intercorrelations

**Fig. 2:**
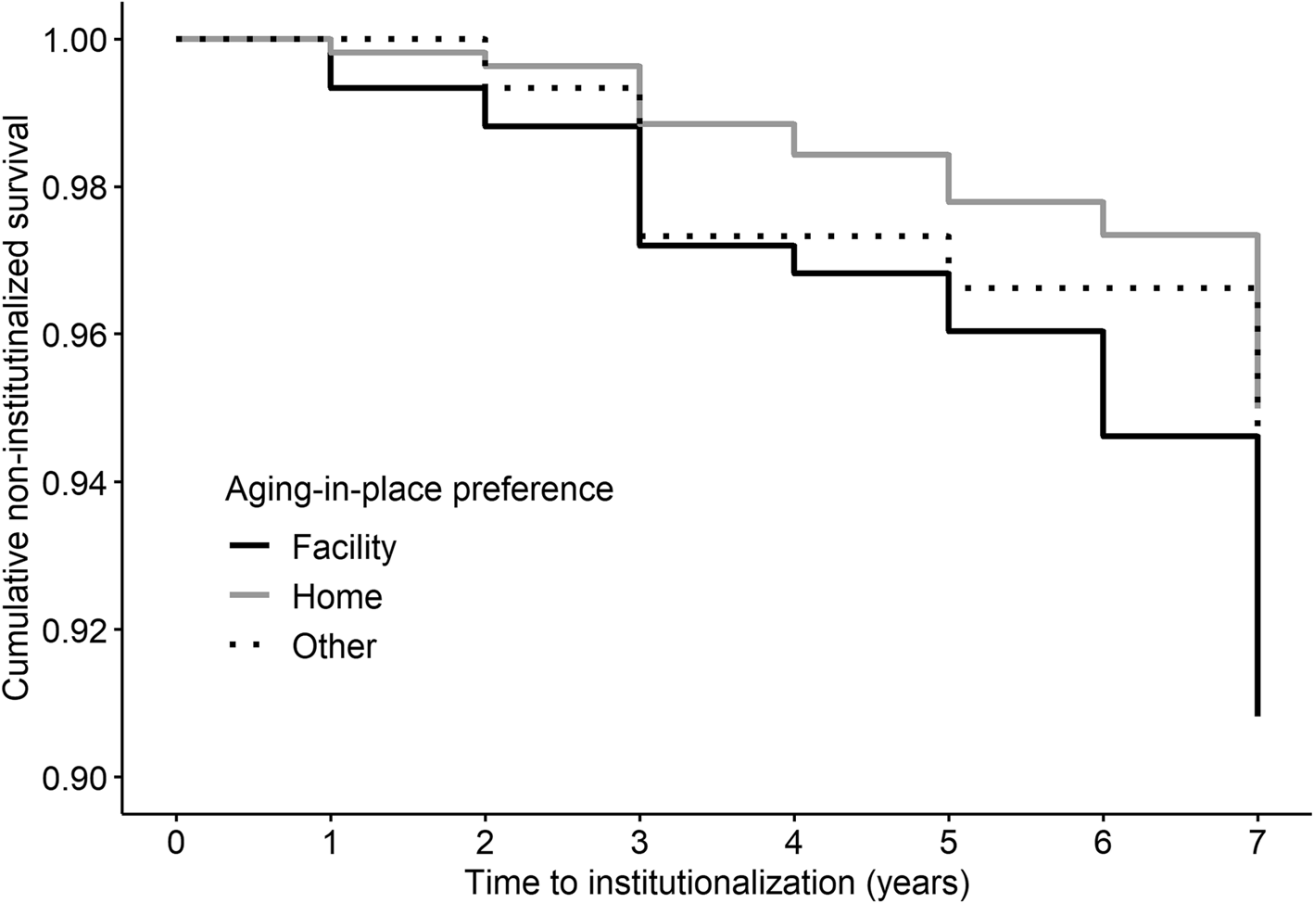
Cumulative non-institutionalized survival stratified by aging-in-place preferences if confined to bed

Participants were followed up for an average of 6.21 years (*SD* = 1.64). During the seven-year period, 78 respondents (6.0%) were institutionalized. Of these, 43 respondents were admitted to long-term hospitals, and 35 respondents were admitted to long-term care facilities. Fig. 2 visualizes cumulative non-institutionalized survival stratified by the desired place of care if confined to bed, using the Kaplan–Meier methods, indicating that survival curves did not intersect each other. Also, the associations between the Schoenfeld residuals and time to institutionalization were not statistically significant (*p*s > 0.617). As a result, the proportional hazard assumptions were not violated.

Table 1 shows descriptive statistics of the observed sample by institutionalization (*N* = 1,290). If confined to bed, 48.7% of the total respondents preferred to stay in long-term care facilities, whereas 39.6% preferred to stay at home. A detailed breakdown of the place of care desired by the individuals indicated was as follows: the respondents preferring to live in long-term care facilities (*N* = 597) chose hospital (*n* = 419) or nursing home (*n* = 178); (ii) those preferring to live in their homes (*N* = 542) chose home with informal caregivers (*n* = 234), home with formal caregivers (*n* = 277), or retirement housing (*n* = 31); and (iii) the remaining respondents (*N* = 151) chose other (*n* = 9) or do not know (*n* = 142).

**Table 1:** Descriptive statistics of the observed sample

Variables	Total		Non-institutionalized		Institutionalized		Range
	*n*	*M* (*SD*) / %	*n*	*M* (*SD*) / %	*n*	*M* (*SD*) / %	
Age (years)	1,290	75.68 (4.80)	1,212	75.51 (4.70)	78	78.38 (5.49)	70—98
Gender (% female)	1,290	58.8	1,212	57.9	78	71.8	
Education (years)	1,268	9.09 (2.65)	1,193	9.14 (2.66)	75	8.23 (2.25)	0—17
Perceived financial status	1,190	2.70 (1.00)	1,121	2.72 (1.00)	69	2.38 (1.06)	0—4
Living arrangement (% co-residing)	1,290	84.3	1,212	84.7	78	76.9	
Marital status (% married)	1,290	56.8	1,212	57.8	78	41.0	
Co-resident children (% yes)	1,290	47.7	1,212	47.4	78	51.3	
Non-coresident children (% yes)	1,290	86.1	1,212	86.5	78	80.8	
Physical function	1,277	38.94 (3.86)	1,200	39.07 (3.47)	77	36.92 (7.50)	2—40
Cognitive function	1,000	7.73 (1.38)	941	7.79 (1.32)	59	6.73 (1.83)	1—9
Aging-in-place preferences (% yes)	1,290		1,212		78		
Facility		48.7		45.4		70.5	
Home		39.6		42.8		29.5	
Other		11.7		11.8		10.3	

Note: *N* = 1,290. Six percent of the respondents were institutionalized during the follow-up

Table S1 presents the intercorrelations between the observed variables (see Additional file 1). The results indicated multicollinearity among family networks: Living arrangement was moderately to strongly correlated with marital status and co-resident children (*r*s = 0.54 and 0.41, respectively). To avoid multicollinearity, we examined the associations of family networks with institutionalization in two models: Model 1 included living arrangements, whereas model 2 included marital status and co-resident children.

### Association of aging-in-place preferences with institutionalization

To check the imputation model, we compared the distributions of the incomplete variables (i.e., education, perceived financial status, physical function, and cognitive function) between the observed and imputed data (see Table S2 for details in Additional file 2). The observed and imputed samples had similar means and standard deviations, ensuring that the obtained results were reliable.

Next, we examined the association of aging-in-place preferences if confined to bed with institutionalization using Cox proportional hazard models. Table 2 then summarizes the results of the estimated models based on the imputed samples. Respondents who preferred to reside in their homes if confined to bed were less likely to be institutionalized than those preferring to reside in long-term care facilities across models (HR = 0.53, 95% CI 0.32–0.87 for crude model; HR = 0.47, 95% CI 0.27–0.79 for model 1 including living arrangement; HR = 0.45, 95% CI 0.27–0.76 for model 2 including marital status and co-resident children). In terms of covariates, older age and physical and cognitive impairment were associated with institutionalization.

**Table 2:** Association of aging-in-place preferences with institutionalization based on the imputed samples

Predictors	Institutionalization^b^	Crude model	Adjusted model 1	Adjusted model 2
		HRs (95% CI)	HRs (95% CI)	HRs (95% CI)
Aging-in-place preferences				
Facility^a^	47 (7.9%)	Ref.	Ref.	Ref.
Home	23 (4.2%)	0.53 (0.32—0.87)*	0.47 (0.27—0.79)**	0.45 (0.27—0.76)**
Other	8 (5.3%)	0.62 (0.29—1.32)	0.53 (0.25—1.12)	0.51 (0.24—1.10)
Age		1.12 (1.08—1.17)***	1.10 (1.05—1.15)***	1.09 (1.05—1.14)***
Gender (ref: male)		1.70 (1.04—2.78)*	1.06 (0.63—1.79)	1.04 (0.59—1.82)
Education (years)		0.87 (0.80—0.96)*	1.00 (0.90—1.10)	1.00 (0.90—1.10)
Perceived financial status		0.74 (0.60—0.92)**	0.85 (0.68—1.06)	0.84 (0.67—1.05)
Living arrangement (ref: living alone)		0.62 (0.37—1.05)	0.74 (0.43—1.29)	―
Marital status (ref: no)		0.51 (0.33—0.80)**	―	0.85 (0.50—1.44)
Co-resident children (ref: no)		1.15 (0.74—1.80)	―	1.10 (0.70—1.73)
Non-coresident children (ref: no)		0.71 (0.40—1.24)	0.74 (0.42—1.32)	0.75 (0.42—1.33)
Physical function		0.92 (0.90—0.95)***	0.95 (0.92—0.98)**	0.95 (0.91—0.98)**
Cognitive function		0.67 (0.59—0.77)***	0.78 (0.66—0.92)**	0.79 (0.67—0.93)**

Note: *P < .05, **P < .01, ***P < .001. *N* = 1,290.

^a^Reference group

^b^The number (ratio in parentheses) of institutionalization during the follow-up was presented according to aging-in-place preferences

*HR* hazard ratio, *CI* confidence interval

To check the robustness of the results reported above, we also conducted the Cox proportional hazard models based on the complete-case sample without missing values on the study variables. Forty-one respondents (4.5%) were institutionalized during the follow-up. As shown in Table S3 (see Additional file 3), we observed a similar, albeit statistically not significant, association of aging-in-place preferences with institutionalization in the models based on the complete-case sample.

## Discussion

This study examined whether individuals preferring to age in place if confined to bed were less likely to be institutionalized (i.e., admitted to a nursing home and hospitalized for a long term) among Japanese older adults. Using nationally representative data collected from 1999 to 2006, we found that, compared to individuals preferring to reside in long-term care facilities if they were confined to bed, those preferring to stay in their homes were less likely to be institutionalized, even after adjusting for relevant covariates. Our findings suggest that individuals’ aging-in-place preferences tend to be considered under the long-term care insurance system when deciding whether to stay at home versus move to a nursing home or a hospital. However, our results also indicate that there are discrepancies between the actual and desired places of receiving care. Indeed, some individuals preferred to stay at home if confined to bed but were institutionalized. This study assessed individual preferences at baseline, but they might change during the follow-up. A further longitudinal examination should be conducted to capture such time-varying preferences.

Our findings have implications for policymakers and clinicians. If older adults are confined to bed and prefer to live at home, adequate care provision from their families and community is required. Still, there are gaps between the desired places for living and dying or between the actual and desired places of death [1, 2]. The Japanese government and local municipalities should continue to facilitate community-based integrated care [24] to enable older care recipients to live at home for as long as possible until death. Furthermore, despite the traditional paternalism [13], shared decision-making between patients and clinicians is now being facilitated in medical settings [25, 26]. Such support for older adults’ care decision-making would also be important in care settings. To our knowledge, only one interventional study plans to directly test the effect of facilitating decision-making for aging-in-place [27]. More studies are needed to promote individual autonomy in care decision-making.

Regarding covariates, we found that older age and physical and cognitive impairment were associated with institutionalization. These findings were consistent with previous studies [3, 4]. In contrast, the role of family networks was inconsistent across studies. Specifically, while earlier studies indicate that living alone is predictive of institutionalization [7], especially among men [9], this study did not observe such an association. There could be several reasons for the inconsistent results. First, the proportion of respondents living alone was relatively small (15.7%), which may have caused this study to be limited in statistical power to detect differences among living arrangements. Yet, given the increasing rates of living alone among Japanese older adults, from 19.7% in 2000 to 26.4% in 2017 [1], more recent cohorts may be more likely to face difficulty continuing to stay at home if confined to bed. Second, this study did not specify detailed family relationships with the participants, except for marital status. Indeed, in the crude model, those having a spouse were more likely to continue to live at home. Spouses might continue caring for their partners even if the caregiver burden became more severe. Conversely, a previous study reported, when daughters-in-law were caregivers, older care recipients were at risk of institutionalization [5]. Third, we did not consider the time-varying nature of family networks. For example, even if older adults lived alone independently at baseline, they could start to need care and live with families. Future research should capture dynamic family networks over time.

This study has strengths, such as a nationally representative sample, a high response rate at baseline, and a long follow-up period. However, there are also limitations to note. First, we assessed institutionalization using self- and proxy-reported data. Objective data sources should be utilized to obtain information about institutionalization. Some respondents might be censored without reporting institutionalization, leading to its relatively low occurrence. In addition, we created a composite outcome by defining institutionalization as nursing home admission and long-term hospitalization. Because of the low occurrence rates, this study lacked sufficient statistical power to examine the association of aging-in-place preferences with the two outcomes separately.

Second, the timing of the baseline survey should be interpreted with caution. In this study, aging-in-place preferences were assessed in 1999. Since 2000, however, individuals’ preferences for the place of care might change after the long-term care insurance system was introduced. Before the implementation of the long-term care insurance system, 43% of older patients stayed in hospitals for more than six months due to the lack of long-term care facilities [28], but the provisions of formal care, including long-term care facilities and home care services, have been enriched in both quantity and quality since then. Indeed, according to a recent study of Japanese older adults [29], the preference for home care services increased before and after the reinforcement of the long-term care insurance system, but the preference for long-term care services remained relatively stable. Therefore, our findings should be updated to reflect more recent data.

Third, the measurement of aging-in-place preferences is not comparable to those in previous studies. Thus, we need to be careful in generalizing the present results and interpreting differences across studies. For instance, another study of Japanese older adults [29] reported that the ratio of respondents preferring to reside in long-term care facilities if confined to bed was 30.1% in 1998. Unlike the present study, however, the measurement did not include a hospital as a long-term care facility.

Fourth, although families play a crucial role in care decision-making, particularly in collectivist cultures [11, 12], the NSJE did not collect data detailed on families. For instance, even if older adults preferred to reside in their homes, their families might not be able to provide sufficient care. Indeed, previous studies suggest that a higher caregiver burden predicts institutionalization among older adults with dementia [30]. Relatedly, we did not utilize the data on formal care because it could mediate the association of aging-in-place preferences with institutionalization. When we included the available variables on home care services (i.e., home-help, short-stay, and day-care services), formal care was not statistically associated with institutionalization (see Table S4 for details in Additional file 4). In contrast, other studies of Japanese older adults reported that home care services, including home-visit nursing services and rental services for assistive devices, were related to continuing to live at home [31, 32], indicating that formal care could compensate for informal care. Also, we did not consider the region of residence (e.g., urban versus rural). Availability of formal care varies according to regions, which may exert an impact on the place of care and death [33]. In the NSJE data, however, only 13 major cities, including Tokyo Metropolitan Area, can be identified.

## Conclusions

This study demonstrated that individuals preferring to reside in their homes were less likely to be institutionalized. Autonomous decision-making has been traditionally less accepted in Japan, but our findings suggest that individuals’ aging-in-place preferences tend to be considered after the implementation of the long-term care insurance system in 2000. Given that decisions are shared and made by individuals, families, and clinicians in collectivistic cultures, such as Asia [11, 12], families should consider older adults’ preferences for place of care whenever possible. Also, clinicians should respect and incorporate such shared decision-making processes when deciding where individuals are to receive care.

## Supplementary Information

**Additional file 1: Table S1.** Intercorrelations among the study variables based on the complete sample.

**Additional file 2: Table S2.** Descriptive statistics of the observed and imputed samples for the incomplete variables.

**Additional file 3: Table S3.** Association of aging-in-place preferences with institutionalization based on the complete-case sample.

**Additional file 4: Table S4.** Association of aging-in-place preferences and home care services with institutionalization based on the imputed samples.

## Data Availability

The NSJE datasets analysed during the current study are publicly available in the Social Science Japan Data Archive repository, https://ssjda.iss.u-tokyo.ac.jp/Direct/gaiyo.php?lang=eng&eid=0823 and https://ssjda.iss.u-tokyo.ac.jp/Direct/gaiyo.php?eid=1185.
