# Supplementary material for: Aging-in-place preferences and institutionalization among Japanese older adults: a 7-year longitudinal study
Source: BMC Geriatr. 2022 Jan 21;22:66. doi: 10.1186/s12877-022-02766-5 (PMC8780808; doi:10.1186/s12877-022-02766-5)
Supplement: Supplementary file 1 — Additional file 1: Table S1. Intercorrelations among the study variables based on the complete sample. [file 12877_2022_2766_MOESM1_ESM.docx]

**Table S1.** Intercorrelations among the study variables based on the complete sample.

| Variables | 2 | 3 | 4 | 5 | 5 | 6 | 7 | 9 | 10 | 11 | 12 |
| --- | --- | --- | --- | --- | --- | --- | --- | --- | --- | --- | --- |
| 1. Age (years) | .07* | —.21*** | .02 | —.06 | —.25*** | .09** | .03 | —.11*** | —.27*** | .05 | —.04 |
| 2. Gender (ref: male) |  | —.21*** | —.06 | —.22*** | —.45*** | —.04 | —.07* | —.03 | —.18*** | —.11*** | —.01 |
| 3. Education (years) |  |  | .16*** | .03 | .17*** | —.13*** | .02 | .11*** | .31*** | —.03 | .03 |
| 4. Perceived financial status |  |  |  | .12*** | .08* | .07* | .06 | .13*** | .09** | .11** | —.02 |
| 5. Living arrangement (ref: living alone) |  |  |  |  | .54*** | .41*** | .14*** | .03 | .00 | .17*** | .01 |
| 6. Marital status (ref: no) |  |  |  |  |  | .04 | .14*** | .06 | .15*** | .13*** | .03 |
| 7. Co-resident children (ref: no) |  |  |  |  |  |  | .02 | —.03 | —.03 | .14*** | .05 |
| 8. Non-coresident children (ref: no) |  |  |  |  |  |  |  | .03 | .02 | .09** | .02 |
| 9. Physical function |  |  |  |  |  |  |  |  | .24*** | —.04 | .04 |
| 10. Cognitive function |  |  |  |  |  |  |  |  |  | —.05 | .02 |
| Aging-in-place preference (ref: facility) |  |  |  |  |  |  |  |  |  |  |  |
| 11. Home |  |  |  |  |  |  |  |  |  |  | —.31*** |
| 12. Other |  |  |  |  |  |  |  |  |  |  |  |

Note: **P* < .05, ***P* < .01, ****P* < .001. *N* = 904.
