# Supplementary material for: Aging-in-place preferences and institutionalization among Japanese older adults: a 7-year longitudinal study
Source: BMC Geriatr. 2022 Jan 21;22:66. doi: 10.1186/s12877-022-02766-5 (PMC8780808; doi:10.1186/s12877-022-02766-5)
Supplement: Supplementary file 2 — Additional file 2: Table S2. Descriptive statistics of the observed and imputed samples for the incomplete variables. [file 12877_2022_2766_MOESM2_ESM.docx]

**Table S2.** Descriptive statistics of the observed and imputed samples for the incomplete variables.

|  | The observed sample | | The imputed samples | |
| --- | --- | --- | --- | --- |
| Variables | *n* | *M* (*SD*) | *n* | *M* (*SD*) |
| Education | 1,268 | 9.09 (2.65) | 22 | 9.08 (2.65) |
| Perceived financial status | 1,190 | 2.70 (1.00) | 100 | 2.70 (1.00) |
| Physical function | 1,277 | 38.94 (3.86) | 13 | 38.93 (3.87) |
| Cognitive function | 1,000 | 7.73 (1.38) | 290 | 7.73 (1.38) |

Note: The values were calculated based on pooled data over 30 imputations.
