# Supplementary material for: Aging-in-place preferences and institutionalization among Japanese older adults: a 7-year longitudinal study
Source: BMC Geriatr. 2022 Jan 21;22:66. doi: 10.1186/s12877-022-02766-5 (PMC8780808; doi:10.1186/s12877-022-02766-5)
Supplement: Supplementary file 3 — Additional file 3: Table S3. Association of aging-in-place preferences with institutionalization based on the complete-case sample. [file 12877_2022_2766_MOESM3_ESM.docx]

**Table S3.** Association of aging-in-place preferences with institutionalization based on the complete-case sample.

|  | Institutionalization^‡^ | Crude model | Adjusted model 1 | Adjusted model 2 |
| --- | --- | --- | --- | --- |
| Predictors |  | HRs (95% CI) | HRs (95% CI) | HRs (95% CI) |
| Aging-in-place preferences |  |  |  |  |
| Facility^†^ | 28 (6.7%) | Ref. | Ref. | Ref. |
| Home | 18 (4.7%) | 0.70 (0.39—1.26) | 0.60 (0.31—1.15) | 0.56 (0.29—1.08) |
| Other | 5 (4.9%) | 0.69 (0.27—1.78) | 0.72 (0.28—1.88) | 0.71 (0.27—1.86) |
| Age |  | 1.13 (1.08—1.18)*** | 1.10 (1.04—1.17)*** | 1.10 (1.04—1.17)*** |
| Gender (ref: male) |  | 1.98 (1.06—3.72)* | 1.33 (0.68—2.61) | 1.33 (0.64—2.78) |
| Education (years) |  | 0.87 (0.80—0.96)* | 1.00 (0.89—1.12) | 1.00 (0.89—1.12) |
| Perceived financial status |  | 0.76 (0.59—0.98)* | 0.88 (0.67—1.15) | 0.86 (0.66—1.12) |
| Living arrangement (ref: living alone) |  | 0.54 (0.29—1.00)* | 0.70 (0.36—1.34) | ― |
| Marital status (ref: no) |  | 0.47 (0.27—0.82)** | ― | 0.87 (0.45—1.68) |
| Co-resident children (ref: no) |  | 1.04 (0.60—1.81) | ― | 1.11 (0.63—1.95) |
| Non-coresident children (ref: no) |  | 0.68 (0.34—1.36) | 0.74 (0.37—1.50) | 0.74 (0.37—1.51) |
| Physical function |  | 0.92 (0.89—0.95)*** | 0.94 (0.90—0.98)** | 0.94 (0.90—0.97)*** |
| Cognitive function |  | 0.62 (0.53—0.73)*** | 0.75 (0.62—0.91)** | 0.76 (0.63—0.92)** |

Note: **P* < .05, ***P* < .01, ****P* < .001. *N* = 904. HR = hazard ratio; CI = confidence interval.

^†^Reference group.

^‡^ The number (ratio in parentheses) of institutionalization during the follow-up was presented according to aging-in-place preferences.
