# Supplementary material for: Aging-in-place preferences and institutionalization among Japanese older adults: a 7-year longitudinal study
Source: BMC Geriatr. 2022 Jan 21;22:66. doi: 10.1186/s12877-022-02766-5 (PMC8780808; doi:10.1186/s12877-022-02766-5)
Supplement: Supplementary file 4 — Additional file 4: Table S4. Association of aging-in-place preferences and home care services with institutionalization based on the imputed samples. [file 12877_2022_2766_MOESM4_ESM.docx]

**Table S4.** Association of aging-in-place preferences and home care services with institutionalization based on the imputed samples.

|  | Crude model | Adjusted model 1 | Adjusted model 2 |
| --- | --- | --- | --- |
| Predictors | HRs (95% CI) | HRs (95% CI) | HRs (95% CI) |
| Aging-in-place preferences |  |  |  |
| Facility^†^ | Ref. | Ref. | Ref. |
| Home | 0.53 (0.32—0.87)* | 0.47 (0.27—0.79)** | 0.45 (0.27—0.77)** |
| Other | 0.62 (0.29—1.32) | 0.53 (0.25—1.13) | 0.51 (0.24—1.10) |
| Home care services (ref: no) | 2.89 (1.44—5.79)** | 1.25 (0.57—2.77) | 1.30 (0.59—2.88) |

Note: **P* < .05, ***P* < .01. *N* = 1,290. HR = hazard ratio; CI = confidence interval. Sixty-two respondents (4.8%) utilized home care services. Other covariates were included in the models, but the results were not shown.

^†^Reference group.
